# Global trends of esophageal cancer among individuals over 60 years: an epidemiological analysis from 1990 to 2050 based on the global burden of disease study 1990-2021

**DOI:** 10.3389/or.2025.1616080

**Published:** 2025-10-09

**Authors:** Simiao Lu, Kexun Li, Kangning Wang, Guangyuan Liu, Yongtao Han, Lin Peng, Lei Chen, Xuefeng Leng

**Affiliations:** ^1^ Department of Thoracic Surgery, Sichuan Clinical Research Center for Cancer, Sichuan Cancer Hospital & Institute, Sichuan Cancer Center, University of Electronic Science and Technology of China (UESTC), Chengdu, China; ^2^ School of Public Health, Chongqing Medical University, Chongqing, China; ^3^ Department of Thoracic Surgery I, Key Laboratory of Lung Cancer of Yunnan Province, Yunnan Cancer Hospital, The Third Affiliated Hospital of Kunming Medical University (Yunnan Cancer Hospital), Kunmung, Yunnan; ^4^ Department of Oncology and Cancer Institute, Sichuan Academy of Medical Sciences, Sichuan Provincial People’s Hospital, University of Electronic Science and Technology of China

**Keywords:** esophageal cancer, global burden of disease, management, incidence, mortality

## Abstract

**Objective:**

Esophageal cancer (EC) ranks as the sixth leading cause of cancer-related deaths globally, with over 500,000 new cases annually. Understanding trends in individuals over 60 is critical for enhancing treatment and the success of early diagnosis and screening.

**Materials and methods:**

This study analyzed global, regional, and national trends in EC management among individuals aged 60 years and older, spanning from 1990 to 2021, utilizing data from the Global Burden of Disease Study 2021. We employed the integrating differential equations to enhance the accuracy of incidence, prevalence, mortality, and Disability-Adjusted Life Years (DALYs). The Bayesian Age-Period-Cohort (BAPC) model was also used to forecast future trends up to 2050.

**Results:**

Our findings indicate significant shifts in the EC burden among those over 60, with a notable increase in absolute numbers from 1990 to 2021, despite a decline in age-standardized rates. The incidence rose by 185%, while the age-standardized prevalence rate decreased by 17.02%. Socioeconomic factors, indicated by the Social Demographic Index, revealed varying trends across different regions and income levels, highlighting the influence of economic status on EC outcomes.

**Conclusion:**

Analysis indicates varying trends across different regions. Behavioral risk factors, particularly smoking and alcohol use, significantly contribute to the burden of EC, especially among males. Projections suggest that despite declining age-standardized rates, the absolute number of cases, deaths, and DALYs will continue to rise due to population growth and aging, highlighting the ongoing global challenge of EC.

## Introduction

Esophageal cancer (EC) represents a significant global health challenge, ranking as the sixth leading cause of cancer-related deaths worldwide. With over 500,000 new cases diagnosed annually, this malignancy poses a formidable burden on healthcare systems globally (1–3). In high-income countries, significant strides have been made in reducing the burden of esophageal cancer through advancements in early detection, surgical techniques, and targeted therapies. These improvements have led to enhanced survival rates and a decrease in incidence within these regions. Conversely, low- and middle-income countries face persistent challenges, including late-stage diagnoses and limited access to advanced treatments, which hinder their ability to achieve similar progress (4–6).

Furthermore, esophageal cancer can be categorized into two primary histological subtypes: squamous cell carcinoma (SCC) and adenocarcinoma. In east Asia such as China and Japan, SCC predominates, accounting for approximately 90% of esophageal cancer cases, while adenocarcinoma is more prevalent in the United States and Europe, representing around 70% of cases (1–3). For resectable esophageal cancer, treatment generally involves a combination of surgery, radiation therapy, chemotherapy, and immunotherapy. Early-stage tumors confined to the mucosa and less than 200 μm in depth may be managed with endoscopic submucosal dissection (ESD) (7–10). However, for advanced-stage esophageal cancer, traditional treatments often yield suboptimal results. Surgical intervention can be challenging due to extensive tumor spread, and while chemotherapy and radiation therapy may alleviate symptoms, their impact on overall survival is limited, with 5-year survival rates ranging from approximately 15%–25% (11,12). Hence, early diagnosis and treatment are crucial for controlling disease progression and minimizing unnecessary healthcare resource utilization. As the global population continues to age, the incidence of EC is projected to rise, highlighting the urgent need for effective management strategies tailored to this demographic. The Global Burden of Disease (GBD) study, a comprehensive and systematic assessment of health metrics, provides valuable insights into the trends and patterns of EC over the past three decades (13–16).

Understanding these disparities and advancements is essential for developing effective global health strategies. This study emphasizes the need for international collaboration to enhance screening programs, ensure equitable access to healthcare, and promote research into innovative therapeutic approaches targeting individuals over 60 years of age. To better understand the global trends and disparities in esophageal cancer, this study utilizes robust analytical frameworks, specifically the Bayesian Age-Period-Cohort model. These models provide a comprehensive analysis of disease incidence, prevalence, and mortality. By addressing these challenges, the global community can strive to reduce the overall burden of esophageal cancer and improve patient outcomes worldwide.

## Methods

### Study population and data collection

This study investigates the global, regional, and national advancements in the management of EC by employing a comprehensive data synthesis from the GBD Study 2021. We incorporated differential equations to process diverse epidemiological data, thereby enhancing the accuracy of statistics related to incidence, prevalence, mortality, and Disability-Adjusted Life Years (DALYs). The study population consisted of individuals aged 60 years and older who were diagnosed with esophageal cancer. The data spanned from 1990 to 2021 and included age, sex, and location-specific statistics across 204 countries and territories. These were categorized into 21 GBD regions based on geographic proximity and further classified into quintiles according to the Social Demographic Index (SDI), a composite measure that assesses socio-economic status through *per capita* income, educational attainment, and fertility rates.

### Statistical analysis

This study examines the dataset structure by calculating counts and rates for key measures. Age-standardized rates (ASRs) per 100,000 were computed using the World Health Organization’s world standard population. Through joinpoint regression analysis, we identified significant trend changes and calculated annual percentage changes (APCs) and average annual percentage change (AAPC) to assess temporal trends from 1990 to 2021. To investigate the relationship between socio-economic status and EC indicators, we employed Pearson correlation and frontier analysis. These methods evaluated the strength of linear relationships and established benchmarks by comparing the EC burden across countries while adjusting for socio-economic status. Additionally, we utilized the Bayesian Age-Period-Cohort (BAPC) model to forecast future trends in EC incidence, prevalence, mortality, and DALYs up to 2050. This advanced statistical modeling accounted for age, period, and cohort effects, integrating past data and probability distributions to estimate future patterns of EC. Our methodology adheres to the strengthened reporting guidelines of cohort, cross-sectional, and case-control studies in epidemiology and the STROCSS guidelines (17–19). All statistical analyses were executed using R version 4.2.3, and results were reported with 95% confidence intervals, where p-values less than 0.05 were deemed statistically significant.

### Ethical considerations

As this study utilized publicly available data from the Global Burden of Disease study (http://ghdx.healthdata.org/gbd-results-tool) (16), no ethical approval was required for the analysis. All data were anonymized and aggregated, ensuring patient confidentiality.

## Results

### Global trends

**FIGURE 1:**
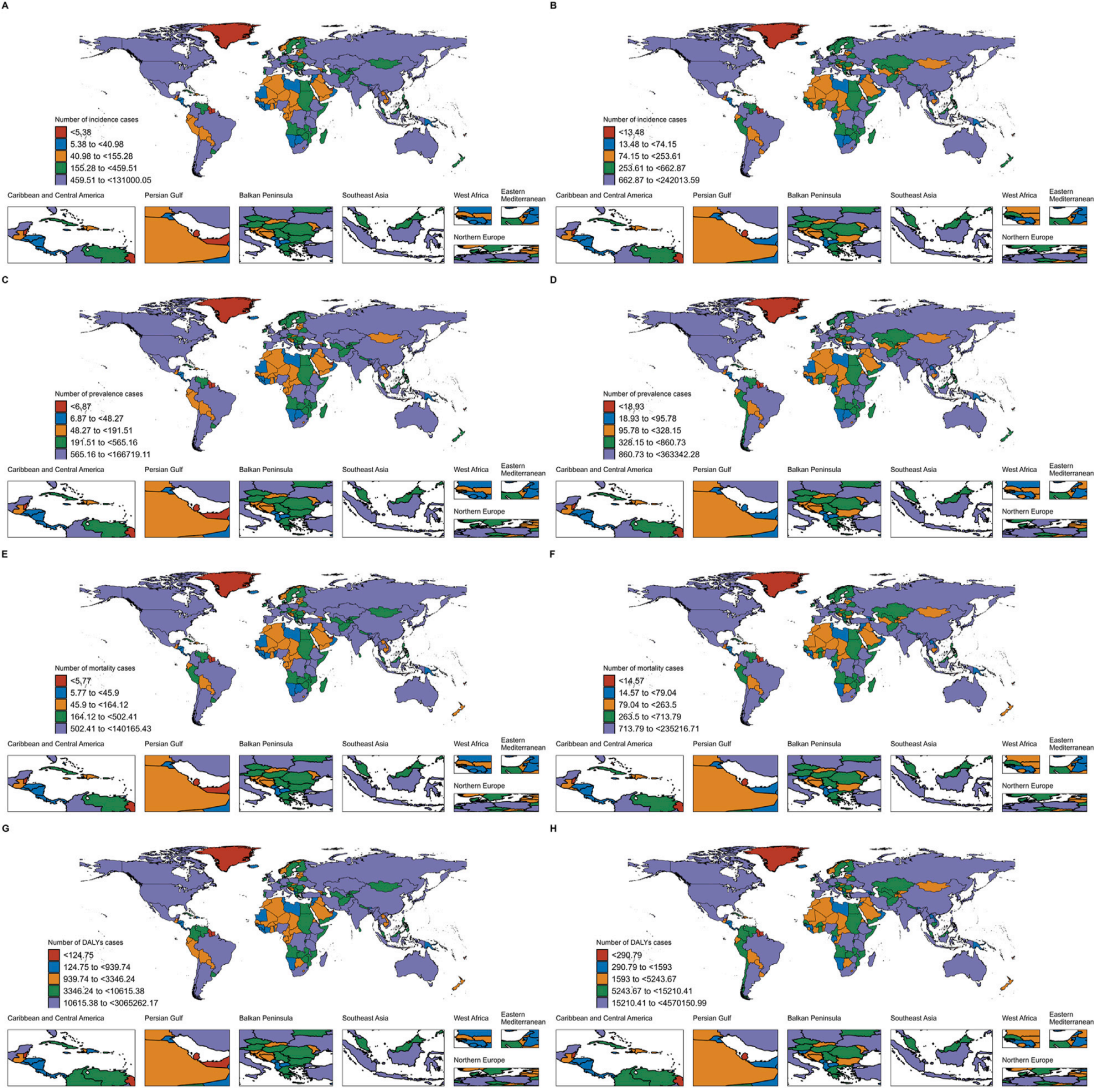
Incidence, Prevalence, Mortality and DALYs of EC in 204 countries and territories. **(A)** Incidence of EC in 204 countries and territories in 1990; **(B)** Incidence of EC in 204 countries and territories in 2021; **(C)** Prevalence of EC in 204 countries and territories in 1990; **(D)** Prevalence of EC in 204 countries and territories in 2021; **(E)** Mortality of EC in 204 countries and territories in 1990; **(F)** Mortality of EC in 204 countries and territories in 2021; **(G)** DALYs of EC in 204 countries and territories in 1990; **(H)** DALYs of EC in 204 countries and territories in 2021.

The investigation into the global trends of EC over 60 from 1990 to 2021 reveals significant shifts in both the incidence and prevalence of the disease. During this period, the number of patients incidence with EC over 60 increased by 185%, rising from 0.23 million cases in 1990 to 0.42 million cases in 2021. Despite this increase in absolute numbers, the age-standardized prevalence rate of EC among the global population decreased by 17.02%, from 47 per 100,000 in 1990 to 39 per 100,000 in 2021, reflecting an average annual decline of −0.63% (Figure 1; Table 1).

**TABLE 1:** Incidence and Prevalence of EC in people aged ≥60 years at global and regional level, 1990-2021.

Location	Incidence		Prevalence		Mortality		DALYs	
	Number of cases (95% UI)		Number of cases (95% UI)		Number of cases (95% UI)		Number of cases (95% UI)	
	1990	2021	1990	2021	1990	2021	1990	2021
Global	226,079.17 (249,281.20–198,590.03)	419,061.14 (475,708.15–364,202.99)	317.08 (377.84–263.74)	654,079.55 (739,407.63–572,182.66)	238,354.31 (262,763.27–209,145.63)	407,757.52 (462,820.18–354,290.58)	5,119,495.75 (5,661,083.95–4,494,140.26)	8,033,323.87 (9,134,090.17–7,010,148.16)
High SDI	42,317.25 (44,030.04–39,848.44)	82,769.82 (87,755.69–74,939.66)	1,302.48 (1,470.95–1,145.44)	168,745.37 (179,205.93–153,844.91)	40,860.53 (42,570.08–38,501.18)	71,034.87 (75,456.20–64,166.76)	818,101.38 (850,424.32–778,819.43)	1,316,127.05 (1,392,091.11–1,211,600.19)
High-middle SDI	71,543.54 (80,616.04–61,741.53)	130,349.05 (159,751.20–105,690.91)	843.05 (927.51–764.09)	199,024.86 (244,553.95–160,917.99)	76,721.78 (86,358.26–66,166.07)	125,880.51 (153,170.19–102,643.42)	1,644,974.90 (1,854,630.72–1,420,210.36)	2,463,521.46 (3,015,818.51–2,009,083.85)
Middle SDI	88,832.81 (103,277.36–72,467.59)	158,008.96 (190,864.70–130,233.72)	4,819.65 (5,158.23–4,483.44)	227,039.98 (275,446.37–185,917.36)	95,238.27 (110,494.45–77,937.66)	158,449.65 (190,238.81–130,706.67)	2,092,433.32 (2,437,633.76–1,707,268.58)	3,139,735.99 (3,775,812.49–2,592,450.89)
Low-middle SDI	14,379.44 (16,667.34–12,706.63)	31,507.42 (36,604.25–28,111.45)	3,221.35 (3,393.62–3,044.19)	39,055.45 (45,276.32–34,887.22)	15,725.47 (18,271.56–13,898.04)	34,407.75 (40,140.05–30,682.01)	344,393.88 (398,913.25–305,527.51)	724,906.13 (842,157.59–647,910.40)
Low SDI	8,909.78 (10,122.41–7,397.37)	16,262.12 (18,915.39–13,722.03)	1,646.17 (1744.41–1,548.40)	19,983.27 (23,281.42–16,828.49)	9,703.54 (11,023.67–8,057.06)	17,813.58 (20,754.40–15,025.35)	217,437.03 (246,861.38–180,324.25)	385,559.24 (449,398.50–324,774.75)
Andean Latin America	276.31 (329.42–229.99)	619.40 (787.08–487.69)	1,660.70 (2,188.13–1,199.00)	724.42 (927.11–566.05)	313.89 (375.34–261.54)	696.40 (883.13–549.75)	6,031.13 (7,227.48–5,018.32)	12,630.97 (16,083.38–9,918.69)
Australasia	857.64 (961.38–755.77)	1832.74 (2,129.95–1,528.80)	169,232.27 (197,432.32–136,004.41)	3,098.52 (3,629.77–2,594.18)	851.24 (955.31–750.78)	1742.86 (2028.54–1,452.80)	16,683.34 (18,684.79–14,770.33)	30,694.51 (35,653.58–25,918.33)
Caribbean	702.29 (773.57–635.81)	1,261.49 (1,457.12–1,078.09)	10,091.68 (10,489.74–9,674.14)	1,629.08 (1886.16–1,387.87)	777.66 (857.80–704.30)	1,357.85 (1,569.24–1,160.64)	15,244.01 (16,841.84–13,801.92)	27,247.35 (31,540.28–23,229.48)
Central Asia	3,947.21 (4,232.11–3,654.56)	2,416.46 (2,712.92–2,141.82)	7,202.98 (8,394.53–5,872.37)	3,011.99 (3,380.44–2,671.48)	4,358.37 (4,676.89–4,028.39)	2,637.81 (2,960.28–2,337.51)	92,811.61 (99,371.13–86,212.12)	56,976.22 (64,023.52–50,460.95)
Central Europe	2,629.57 (2,770.80–2,481.67)	4,227.07 (4,593.50–3,832.20)	299,679.28 (329,613.28–263,818.20)	5,679.56 (6,185.38–5,156.89)	2,881.13 (3,035.99–2,719.59)	4,500.37 (4,886.71–4,080.81)	60,762.30 (64,005.59–57,454.32)	93,667.52 (101,743.39–85,197.28)
Central Latin America	1,412.10 (1,496.71–1,323.27)	2,739.46 (3,105.70–2,401.23)	67,592.14 (70,504.65–63,921.34)	3,332.69 (3,786.49–2,922.23)	1,593.08 (1,688.12–1,491.47)	3,034.30 (3,431.63–2,658.37)	30,690.89 (32,497.05–28,886.67)	57,559.81 (65,394.15–50,484.66)
Central Sub-Saharan Africa	1,353.22 (1776.90–983.06)	2,482.62 (3,335.93–1767.00)	21,439.73 (23,283.71–19,578.97)	3,106.86 (4,169.10–2,209.60)	1,463.14 (1923.82–1,063.02)	2,693.50 (3,630.42–1913.29)	33,720.56 (44,384.05–24,458.50)	60,568.59 (81,253.55–42,976.87)
East Asia	132,834.63 (154,569.03–107,511.66)	245,248.71 (300,371.53–195,809.51)	15,937.86 (16,570.72–15,048.46)	369,249.00 (453,459.36–294,618.56)	142,059.19 (165,038.95–115,287.24)	237,998.75 (290,930.01–189,676.05)	3,108,490.01 (3,628,315.88–2,519,966.26)	4,642,654.59 (5,706,911.74–3,689,116.56)
Eastern Europe	7,819.02 (8,131.88–7,477.96)	7,331.53 (8,005.48–6,611.21)	90,429.89 (101,880.85–77,924.49)	10,457.27 (11,437.43–9,443.96)	8,255.64 (8,575.22–7,902.01)	7,359.02 (8,026.83–6,654.24)	181,100.07 (188,105.57–173,956.89)	162,611.01 (177,960.91–146,934.94)
Eastern Sub-Saharan Africa	5,965.30 (6,954.16–4,871.98)	10,759.98 (13,050.59–8,875.99)	10,787.85 (12,251.89–8,940.04)	13,263.84 (16,153.52–10,929.36)	6,495.64 (7,582.09–5,309.67)	11,768.39 (14,253.61–9,725.29)	145,548.01 (169,816.48–118,888.46)	256,045.07 (310,599.71–211,693.94)
High-income Asia Pacific	9,517.48 (10,099.00–8,841.95)	22,334.07 (24,236.29–19,650.65)	17,541.23 (20,276.14–15,542.82)	64,612.00 (70,256.29–57,205.90)	7,645.87 (8,112.54–7,103.68)	15,064.12 (16,293.27–13,224.77)	156,115.62 (165,549.01–146,107.78)	261,851.39 (282,433.56–234,601.04)
High-income North America	10,551.29 (10,993.58–9,890.41)	21,714.81 (22,950.64–19,843.42)	113,208.17 (131,865.90–91,447.86)	36,161.77 (38,212.05–33,366.88)	10,051.21 (10,480.10–9,411.11)	19,648.62 (20,789.59–17,941.36)	203,477.21 (211,223.48–192,898.75)	381,818.86 (401,442.69–354,823.27)
North Africa and Middle East	2,653.90 (3,080.26–2,148.48)	5,694.87 (6,440.23–4,874.38)	3,412.43 (3,945.12–2,763.36)	8,936.96 (10,118.82–7,663.40)	2,904.84 (3,377.77–2,352.72)	6,141.36 (6,949.79–5,243.32)	62,305.23 (72,333.86–50,328.94)	122,150.26 (138,540.37–104,544.18)
Oceania	35.18 (48.06–25.78)	76.55 (101.87–58.55)	43.65 (59.79–31.85)	95.82 (127.53–73.25)	37.94 (51.79–28.06)	82.75 (110.76–63.52)	860.21 (1,179.87–631.59)	1816.73 (2,436.30–1,392.80)
South Asia	12,498.20 (15,161.59–10,749.91)	30,063.70 (36,354.71–26,232.33)	15,330.95 (18,540.87–13,255.89)	37,216.54 (44,870.26–32,548.40)	13,624.45 (16,597.03–11,681.36)	32,817.91 (39,775.99–28,644.01)	302,669.90 (367,555.15–260,912.99)	690,293.94 (832,171.12–604,038.43)
Southeast Asia	3,991.02 (4,779.77–3,284.16)	9,824.82 (11,569.55–8,271.53)	4,994.78 (5,990.69–4,109.03)	13,420.32 (15,869.04–11,230.23)	4,326.78 (5,165.39–3,561.23)	10,222.06 (12,033.93–8,626.34)	94,646.77 (113,111.57–77,748.98)	218,615.37 (258,007.93–184,627.54)
Southern Latin America	2,446.51 (2,724.41–2,178.93)	2,764.57 (3,141.54–2,391.39)	2,887.73 (3,210.53–2,583.76)	3,401.74 (3,869.43–2,957.27)	2,718.07 (3,028.89–2,419.52)	3,012.69 (3,423.53–2,598.56)	52,731.53 (58,774.80–47,152.44)	54,853.53 (62,365.07–47,648.61)
Southern Sub-Saharan Africa	1,617.43 (1944.18–1,376.86)	3,732.99 (4,195.99–3,294.16)	1957.48 (2,357.84–1,666.29)	4,511.09 (5,065.83–3,993.95)	1793.12 (2,160.10–1,525.95)	4,084.04 (4,590.79–3,601.46)	37,241.19 (44,712.13–31,751.66)	86,625.64 (97,272.52–76,619.35)
Tropical Latin America	3,553.85 (3,763.70–3,304.44)	8,048.05 (8,632.35–7,298.83)	4,358.80 (4,604.78–4,070.27)	10,315.40 (11,037.69–9,430.80)	3,899.72 (4,133.38–3,625.15)	8,716.83 (9,359.06–7,882.99)	82,624.68 (87,401.11–77,298.85)	180,807.67 (193,340.23–165,246.08)
Western Europe	19,932.05 (20,927.56–18,641.87)	30,868.49 (33,228.70–27,567.37)	27,197.71 (28,597.91–25,577.00)	55,766.26 (60,439.10–50,021.45)	20,668.89 (21,708.40–19,300.50)	28,651.64 (30,752.34–25,567.72)	400,458.51 (419,714.21–377,499.05)	516,599.06 (551,769.00–469,761.04)
Western Sub-Saharan Africa	1,484.99 (1789.61–1,205.48)	5,018.78 (5,997.99–3,708.14)	1780.75 (2,153.86–1,437.35)	6,088.42 (7,308.76–4,477.87)	1,634.44 (1968.78–1,330.77)	5,526.26 (6,600.43–4,099.99)	35,282.99 (42,754.94–28,596.13)	117,235.78 (140,657.73–86,913.61)
Male	149,253.24 (168,012.44–132,620.33)	305,224.02 (355,616.10–259,979.07)	198,706.47 (223,095.14–177,549.00)	468,352.69 (542,422.30–402,843.66)	156,673.948 (176,045.24–139,014.624)	297,229.051 (345,832.705–253,467.92)	3,462,648.64 (3,910,431.13–3,065,291.48)	5,978,539.08 (6,973,734.94–5,090,398.99)
Female	76,825.931 (89,621.841–53,822.682)	113,837.127 (135,645.327–82,817.753)	100,972.808 (117,517.969–70,573.938)	185,726.866 (223,315.866–132,907.621)	81,680.362 (95,157.093–57,149.38)	110,528.467 (131,497.074–81,618.455)	1,656,847.114 (1,943,373.716–1,132,520.185)	2,054,784.792 (2,432,874.454–1,534,957.433)
60–64 years	58,129.75 (64,559.92–51,416.77)	73,708.82 (84,025.07–65,261.57)	94,412.79 (104,486.59–83,767.84)	142,050.43 (161,155.33–126,211.74)	56,532.6 (62,838.51–49,942.82)	65,187.22 (74,287.17–57,786.34)	1,648,101.57 (1,832,464.97–1,456,620.75)	1,901,474.56 (2,169,172.2–1,687,394.43)
65–69 years	56,619.3 (62,688.92–49,067.59)	56,619.3 (62,688.92–49,067.59)	82,238.41 (90,817.81–71,292.22)	168,933.6 (191,673.04–147,546.5)	57,109.5 (63,248.93–49,452.96)	85,434.65 (97,449.83–74,192.94)	1,402,528.9 (1,554,450.19–1,217,271.35)	2,100,376.54 (2,392,290.19–1,823,027.56)
70–74 years	48,736.32 (53,880.84–42,732.71)	93,543.77 (106,349.18–81,690.15)	61,867.61 (68,034.38–54,442.49)	154,469.03 (173,841.31–135,085.08)	51,503.3 (56,994.01–44,852.88)	87,433.46 (99,305.04–76,315.21)	1,041,192.79 (1,153,975.5–907,854.27)	1,770,808.33 (2,015,703.25–1,548,103.3)
75–79 years	34,311.04 (37,626.59–30,513.91)	69,387.43 (78,807.77–60,248.42)	36,962.97 (40,291.04–33,020.99)	96,102.12 (108,879.65–84,200.66)	38,449.89 (42,118.36–34,223.42)	70,165.16 (79,753.4–61,037.06)	623,262.9 (682,685.3–554,267.45)	1,137,272.84 (1,292,062.26–990,870.49)
80–84 years	18,279.85 (19,718.42–16,201.11)	48,802.12 (54,947.82–42,007.04)	16,952.03 (18,175.74–15,066.14)	59,116.2 (66,297.21–51,283.74)	21,815.63 (23,572.22–19,478.89)	52,689.19 (59,442.43–45,378.65)	277,194.87 (299,932.02–247,872.4)	667,867.75 (753,483.53–575,546.31)
85–89 years	8,047.404,853 (8,685.317,776–7,001.496,799)	29,611.72094 (33,373.62693–25,099.99601)	5,998.806,395 (6,453.398,395–5,177.532,517)	26,394.48267 (29,636.91835–22,211.75032)	10,165.62373 (10,984.3258–8,840.811,085)	34,066.13486 (38,208.56007–29,034.50348)	102,849.5082 (111,183.9793–89,560.15899)	343,373.1643 (385,256.8696–292,878.4123)
90–94 years	1955.499,751 (2,121.195,821–1,656.457,467)	9,439.261,474 (10,632.88027–7,743.416,622)	1,246.660,174 (1,354.334,901–1,050.976,016)	1,246.660,174 (1,354.334,901–1,050.976,016)	2,777.781,013 (3,006.906,679–2,353.856,124)	12,781.69252 (14,373.74634–10,545.88177)	24,365.21797 (26,391.98481–20,693.87906)	112,150.6958 (126,121.8677–92,327.65647)
95+ years	332.03 (373.44–260.84)	2,274.59 (2,620.49–1,692.58)	170.14 (191.42–133.47)	1,233.36 (1,423.49–914.51)	499.62 (560.91–393.25)	3,144.66 (3,607.31–2,343.84)	4,138.56 (4,641.62–3,264.87)	25,832.26 (29,694.45–19,265.62)

A gender disparity in EC over 60 incidence is evident, with male patients consistently outnumbering female patients throughout the study period. Specifically, the number of male patients increased from 0.15 million (66.02%) in 1990 to 0.31 million (72.84%) in 2021, while female patients rose from 0.08 million (33.98%) to 0.11 million (27.16%) over the same timeframe. In 1990, males exhibited an age-standardized rate (ASR) of 70 per 100,000, compared to 29 per 100,000 for females. By 2021, the ASR for males had decreased to 63 per 100,000, while females showed a decline to 19 per 100,000 (Tables 1, 2).

**TABLE 2:** Mortality and DALYs of EC in people aged ≥60 years at global and regional level, 1990-2021.

Location	Age-standardized incidence rate (per 100,000)			Age-standardized prevalence rate (per 100,000)			Age-standardized mortality rate (per 100,000)			Age-standardized DALYs rate (per 100,000)		
	1990 (95% UI)	2021 (95% UI)	AAPC (95% UI)	1990 (95% UI)	2021 (95% UI)	AAPC (95% UI)	1990 (95% UI)	2021 (95% UI)	AAPC (95% UI)	1990 (95% UI)	2021 (95% UI)	AAPC (95% UI)
Global	47.25 (52.04–41.49)	39.06 (44.33–33.93)	−0.63 (-0.77 to −0.49)	60.93 (66.95–53.62)	60.21 (68.05–52.65)	−0.04 (-0.17 to 0.09)	50.44 (55.53–44.24)	38.25 (43.41–33.22)	−0.91 (-1.07 to −0.76)	1,040.22 (1,149.30–913.01)	739.86 (841.06–645.41)	−1.12 (-1.26 to −0.97)
High SDI	29.34 (30.54–27.62)	29.50 (31.22–26.88)	0.00 (-0.16–0.17)	47.00 (49.03–44.45)	61.55 (65.24–56.39)	0.84 (0.60–1.09)	28.34 (29.54–26.68)	24.98 (26.49–22.72)	−0.42 (-0.60 to −0.24)	570.41 (593.05–542.94)	483.60 (510.91–447.41)	−0.54 (-0.72 to −0.36)
High-middle SDI	58.02 (65.35–50.02)	51.30 (62.83–41.59)	−0.40 (-0.63 to −0.17)	71.03 (80.01–61.14)	77.54 (95.28–62.67)	0.29 (0.07–0.52)	63.13 (71.01–54.38)	49.80 (60.56–40.59)	−0.78 (-1.02 to −0.55)	1,294.78 (1,459.15–1,116.70)	962.32 (1,177.80–784.67)	−0.97 (-1.23 to −0.71)
Middle SDI	75.67 (87.65–61.89)	49.43 (59.59–40.71)	−1.38 (-1.53 to −1.22)	91.68 (106.53–74.21)	68.63 (83.14–56.17)	−0.91 (-1.05 to −0.78)	82.97 (95.86–68.10)	50.40 (60.37–41.55)	−1.61 (-1.78 to −1.44)	1702.87 (1977.98–1,392.24)	955.41 (1,147.18–788.64)	−1.88 (-2.04 to −1.72)
Low-middle SDI	21.48 (24.97–18.91)	19.08 (22.22–16.97)	−0.37 (-0.54 to −0.20)	24.80 (28.72–21.92)	22.70 (26.36–20.24)	−0.27 (-0.43 to −0.12)	24.06 (28.06–21.18)	21.22 (24.81–18.87)	−0.39 (-0.59 to −0.19)	488.22 (566.94–431.88)	422.18 (491.35–376.81)	−0.46 (-0.62 to −0.30)
Low SDI	35.92 (40.90–29.78)	30.06 (35.02–25.31)	−0.56 (-0.67 to −0.44)	40.91 (46.52–33.89)	34.92 (40.71–29.38)	−0.50 (-0.55 to −0.45)	40.21 (45.80–33.34)	33.77 (39.42–28.44)	−0.54 (-0.66 to −0.43)	827.17 (940.31–686.05)	675.97 (788.11–569.31)	−0.63 (-0.69 to −0.58)
East Asia	133.88 (155.16–108.45)	90.25 (110.36–72.10)	−1.28 (-1.48 to −1.08)	159.64 (185.74–128.40)	131.71 (161.63–105.02)	−0.61 (-0.79 to −0.42)	147.59 (170.72–119.80)	89.05 (108.62–71.05)	−1.63 (-1.85 to −1.42)	2,955.52 (3,439.17–2,397.19)	1,671.14 (2052.44–1,329.33)	−1.85 (-2.05 to −1.64)
Eastern Sub-Saharan Africa	73.47 (85.85–59.89)	61.44 (74.50–50.56)	−0.58 (-0.66 to −0.50)	83.31 (97.22–67.88)	71.34 (86.79–58.72)	−0.51 (-0.55 to −0.46)	82.37 (96.41–67.19)	69.00 (83.61–56.86)	−0.57 (-0.65 to −0.49)	1,688.34 (1971.98–1,378.62)	1,381.79 (1,674.55–1,141.36)	−0.64 (-0.72 to −0.56)
Central Asia	72.17 (77.43–66.74)	26.32 (29.52–23.34)	−3.33 (-4.11 to −2.54)	85.67 (91.71–79.64)	30.99 (34.72–27.52)	−3.31 (-3.96 to −2.65)	80.55 (86.48–74.36)	29.34 (32.89–26.01)	−3.33 (-4.13 to −2.53)	1,647.99 (1764.58–1,529.79)	587.62 (659.17–521.16)	−3.38 (-4.04 to −2.71)
Central Sub-Saharan Africa	57.04 (75.03–41.48)	45.91 (62.35–32.57)	−0.71 (-0.83 to −0.60)	64.27 (84.67–46.49)	52.90 (71.39–37.62)	−0.64 (-0.75 to −0.52)	64.07 (84.52–46.62)	51.62 (70.60–36.54)	−0.71 (-0.82 to −0.60)	1,311.23 (1726.06–952.94)	1,035.06 (1,397.53–734.72)	−0.78 (-0.90 to −0.66)
Southern Sub-Saharan Africa	53.40 (64.30–45.40)	57.42 (64.60–50.47)	0.22 (-0.10–0.53)	61.85 (74.60–52.59)	65.69 (73.80–58.02)	0.19 (-0.21–0.59)	60.38 (72.86–51.32)	64.37 (72.43–56.54)	0.19 (-0.11–0.50)	1,178.01 (1,416.05–1,003.24)	1,264.59 (1,420.84–1,116.27)	0.22 (-0.20–0.64)
Southern Latin America	42.76 (47.63–38.00)	24.36 (27.68–21.08)	−1.80 (-2.01 to −1.58)	49.27 (54.79–44.02)	30.25 (34.42–26.31)	−1.49 (-1.77 to −1.21)	48.08 (53.59–42.68)	26.45 (30.06–22.82)	−1.91 (-2.13 to −1.69)	901.57 (1,005.01–804.97)	487.80 (554.72–423.85)	−1.90 (-2.19 to −1.61)
High-income Asia Pacific	38.17 (40.55–35.33)	35.52 (38.43–31.65)	−0.28 (-0.49 to −0.06)	84.37 (91.74–76.84)	108.05 (117.14–96.57)	0.75 (0.54–0.96)	31.03 (32.95–28.69)	23.07 (24.89–20.56)	−1.02 (-1.10 to −0.94)	614.97 (652.46–574.10)	439.42 (473.58–398.20)	−1.17 (-1.26 to −1.09)
Tropical Latin America	34.41 (36.52–31.79)	25.36 (27.23–22.95)	−0.93 (-1.09 to −0.77)	40.33 (42.67–37.51)	32.01 (34.27–29.21)	−0.68 (-0.87 to −0.50)	38.55 (40.96–35.58)	27.66 (29.72–24.95)	−1.01 (-1.17 to −0.85)	766.04 (811.51–713.67)	561.10 (600.32–511.97)	−0.95 (-1.13 to −0.76)
Western Europe	25.96 (27.26–24.29)	25.00 (26.83–22.56)	−0.13 (-0.36 to 0.10)	35.90 (37.75–33.79)	47.67 (51.54–43.06)	0.92 (0.63–1.21)	26.82 (28.17–25.04)	22.56 (24.14–20.37)	−0.57 (-0.87 to −0.26)	529.84 (555.37–499.70)	439.37 (468.18–403.00)	−0.62 (-0.88 to −0.36)
Australasia	27.93 (31.34–24.56)	25.30 (29.39–21.16)	−0.37 (-0.84 to 0.10)	42.03 (47.49–36.91)	43.73 (51.22–36.71)	0.08 (-0.37–0.54)	27.90 (31.33–24.55)	23.76 (27.65–19.86)	−0.54 (-0.72 to −0.37)	539.75 (604.66–477.31)	434.57 (504.70–367.76)	−0.73 (-0.89 to −0.58)
Caribbean	22.38 (24.65–20.25)	18.97 (21.91–16.21)	−0.44 (-0.63 to −0.26)	26.39 (29.03–23.91)	24.51 (28.38–20.88)	−0.13 (-0.34 to 0.08)	25.03 (27.60–22.65)	20.40 (23.58–17.44)	−0.57 (-0.76 to −0.38)	478.26 (528.33–432.85)	410.00 (474.61–349.56)	−0.42 (-0.61 to −0.22)
High-income North America	22.69 (23.64–21.29)	24.61 (25.99–22.50)	0.20 (0.11–0.29)	34.62 (35.98–32.73)	41.22 (43.54–38.05)	0.53 (0.37–0.68)	21.56 (22.48–20.20)	22.20 (23.47–20.29)	0.06 (-0.10–0.22)	443.82 (460.62–421.09)	435.54 (457.76–405.09)	−0.09 (-0.24 to 0.06)
Eastern Europe	21.38 (22.25–20.40)	15.20 (16.59–13.71)	−0.99 (-1.37 to −0.60)	26.81 (27.87–25.66)	21.44 (23.44–19.37)	−0.59 (-0.97 to −0.21)	22.86 (23.76–21.82)	15.31 (16.69–13.85)	−1.19 (-1.69 to −0.70)	481.15 (499.91–461.53)	333.60 (364.88–301.58)	−1.07 (-1.55 to −0.58)
South Asia	20.14 (24.54–17.20)	17.53 (21.23–15.26)	−0.40 (-0.67 to −0.12)	23.23 (28.18–19.98)	20.79 (25.11–18.16)	−0.35 (-0.60 to −0.10)	22.54 (27.60–19.19)	19.51 (23.69–17.00)	−0.39 (-0.63 to −0.15)	460.54 (561.12–394.98)	386.74 (467.18–338.10)	−0.56 (-0.81 to −0.31)
Central Latin America	15.70 (16.65–14.66)	9.21 (10.43–8.07)	−1.78 (-2.25 to −1.31)	17.60 (18.66–16.51)	10.99 (12.48–9.63)	−1.58 (-2.00 to −1.17)	18.03 (19.12–16.81)	10.28 (11.61–9.00)	−1.86 (-2.33 to −1.40)	328.97 (348.48–308.85)	189.84 (215.52–166.50)	−1.84 (-2.27 to −1.41)
Western Sub-Saharan Africa	15.11 (18.15–12.31)	24.82 (29.55–18.36)	1.62 (1.51–1.73)	17.28 (20.84–13.99)	28.49 (34.09–20.96)	1.63 (1.55–1.71)	16.99 (20.39–13.89)	27.99 (33.29–20.79)	1.63 (1.51–1.74)	343.42 (414.89–279.08)	550.02 (657.63–407.99)	1.54 (1.45–1.62)
North Africa and Middle East	14.57 (16.95–11.81)	11.93 (13.48–10.19)	−0.62 (-0.74 to −0.51)	17.61 (20.39–14.28)	17.82 (20.18–15.24)	0.04 (-0.07–0.15)	16.39 (19.09–13.29)	13.18 (14.90–11.23)	−0.67 (-0.79 to −0.56)	322.34 (374.60–260.84)	242.73 (274.94–207.56)	−0.90 (-1.00 to −0.80)
Southeast Asia	14.25 (17.05–11.74)	13.01 (15.30–10.94)	−0.32 (-0.40 to −0.24)	16.95 (20.31–13.95)	16.90 (19.96–14.14)	−0.02 (-0.10 to 0.06)	15.80 (18.84–13.01)	13.84 (16.27–11.67)	−0.46 (-0.54 to −0.38)	321.73 (384.08–264.54)	275.35 (324.50–232.57)	−0.51 (-0.62 to −0.40)
Central Europe	13.67 (14.41–12.87)	14.14 (15.36–12.81)	0.12 (-0.30–0.54)	16.26 (17.13–15.34)	19.19 (20.90–17.42)	0.57 (0.18–0.96)	15.19 (16.01–14.30)	14.99 (16.28–13.59)	0.01 (-0.31–0.34)	307.11 (323.55–290.00)	317.25 (344.61–288.47)	0.12 (-0.29–0.54)
Andean Latin America	12.18 (14.51–10.14)	8.83 (11.21–6.95)	−0.98 (-1.73 to −0.23)	13.64 (16.24–11.35)	10.21 (13.07–7.99)	−0.76 (-0.97 to −0.54)	13.98 (16.70–11.65)	9.97 (12.64–7.87)	−1.04 (-1.78 to −0.30)	259.57 (310.85–216.06)	178.20 (226.83–140.02)	−1.06 (-1.27 to −0.85)
Oceania	12.05 (16.37–8.92)	10.49 (13.94–8.02)	−0.43 (-0.48 to −0.38)	13.46 (18.34–9.91)	12.07 (16.03–9.23)	−0.37 (-0.41 to −0.32)	13.66 (18.56–10.21)	11.80 (15.75–9.04)	−0.48 (-0.54 to −0.41)	267.00 (363.94–197.76)	229.90 (307.58–176.34)	−0.50 (-0.55 to −0.46)
Male	70.06 (78.55–62.37)	62.61 (72.78–53.38)	−0.38 (-0.52 to −0.24)	88.99 (99.63–79.61)	93.34 (107.92–80.33)	0.14 (0.01–0.28)	75.08 (83.99–66.76)	61.88 (71.83–52.83)	−0.64 (-0.80 to −0.49)	1,551.18 (1746.87–1,375.35)	1,191.5 (1,387.99–1,015.32)	−0.87 (-1.02 to −0.73)
Female	29.13 (33.93–20.51)	19.49 (23.22–14.18)	−1.28 (-1.36 to −1.21)	37.72 (43.86–26.46)	31.79 (38.21–22.76)	−0.52 (-0.63 to −0.41)	31.2 (36.29–21.95)	18.92 (22.50–13.97)	−1.59 (-1.78 to −1.40)	619.00 (725.28–424.56)	352.09 (416.73–263.18)	−1.79 (-1.96 to −1.62)
60–64 years	36.19 (40.20–32.01)	23.03 (26.25–20.39)	−1.43 (−1.63 to −1.24)	58.78 (65.06–52.16)	44.38 (50.35–39.44)	−0.93 (−1.14 to −0.72)	35.20 (39.13–31.10)	20.37 (23.21–18.06)	−1.76 (−1.99 to −1.54)	1,026.16 (1,140.95–906.94)	594.12 (677.77–527.23)	−1.72 (−1.96 to −1.47)
65–69 years	45.81 (60.72–39.70)	34.28 (39.00–29.78)	−0.94 (−1.10 to −0.78)	66.53 (73.47–57.68)	61.24 (69.49–53.49)	−0.28 (−0.49 to −0.08)	46.20 (51.17–40.01)	30.97 (35.33–26.90)	−1.31 (−1.50 to −1.13)	1,134.65 (1,257.55–984.77)	761.44 (867.27–660.90)	−1.29 (−1.46 to −1.12)
70–74 years	57.57 (63.64–50.48)	45.45 (61.67–39.69)	−0.73 (−0.84 to −0.63)	73.08 (80.36–64.31)	75.04 (84.46–65.63)	0.13 (−0.00–0.26)	60.84 (67.32–52.98)	42.48 (48.24–37.08)	−1.13 (−1.23 to −1.03)	1,229.84 (1,363.05–1,072.34)	860.29 (979.26–752.09)	−1.12 (−1.24 to −1.01)
75–79 years	55.74 (61.13–49.57)	52.61 (59.76–45.68)	−0.20 (−0.57 to 0.17)	60.05 (65.45–53.64)	72.87 (82.56–63.84)	0.58 (0.13–1.02)	62.46 (68.42–55.60)	53.20 (60.47–46.28)	−0.53 (−0.92 to −0.14)	1,012.82 (1,109.06–900.44)	862.33 (979.69–751.32)	−0.54 (−0.92 to −0.15)
80–84 years	51.67 (55.74–45.80)	55.72 (62.74–47.96)	0.25 (−0.17–0.67)	47.92 (51.38–42.59)	67.50 (75.70–58.55)	1.14 (0.86–1.42)	61.67 (66.63–55.06)	60.16 (67.87–51.81)	−0.08 (−0.49 to 0.33)	783.57 (847.84–700.68)	762.55 (860.31–657.14)	0.09 (−0.47 to 0.30)
85–89 years	53.29 (57.48–46.33)	64.77 (72.99–54.90)	0.63 (0.43–0.82)	39.70 (42.71–34.26)	57.73 (64.82–48.58)	1.23 (1.01–1.45)	67.27 (72.69–68.51)	74.51 (83.57–63.50)	0.31 (0.10–0.53)	680.62 (735.78–592.68)	751.01 (842.61–640.57)	0.30 (0.09–0.51)
90–94 years	45.63 (49.50–28.66)	52.77 (69.44–43.28)	0.47 (0.26–0.68)	29.09 (31.61–24.53)	39.21 (44.30–31.55)	0.99 (0.80–1.18)	64.82 (70.17–54.93)	71.45 (80.35–58.95)	0.31 (0.09–0.52)	568.59 (615.89–482.92)	626.91 (705.01–516.10)	0.21 (0.09–0.52)
95+ years	32.61 (36.68–25.62)	41.73 (48.08–31.06)	0.81 (0.63–0.99)	16.71 (18.80–13.11)	22.63 (26.12–16.78)	1.00 (0.82–1.18)	49.07 (55.10–38.63)	57.70 (66.19–43.00)	0.54 (0.35–0.73)	406.50 (455.92–320.69)	473.96 (544.82–353.48)	0.51 (0.32–0.71)

**FIGURE 2:**
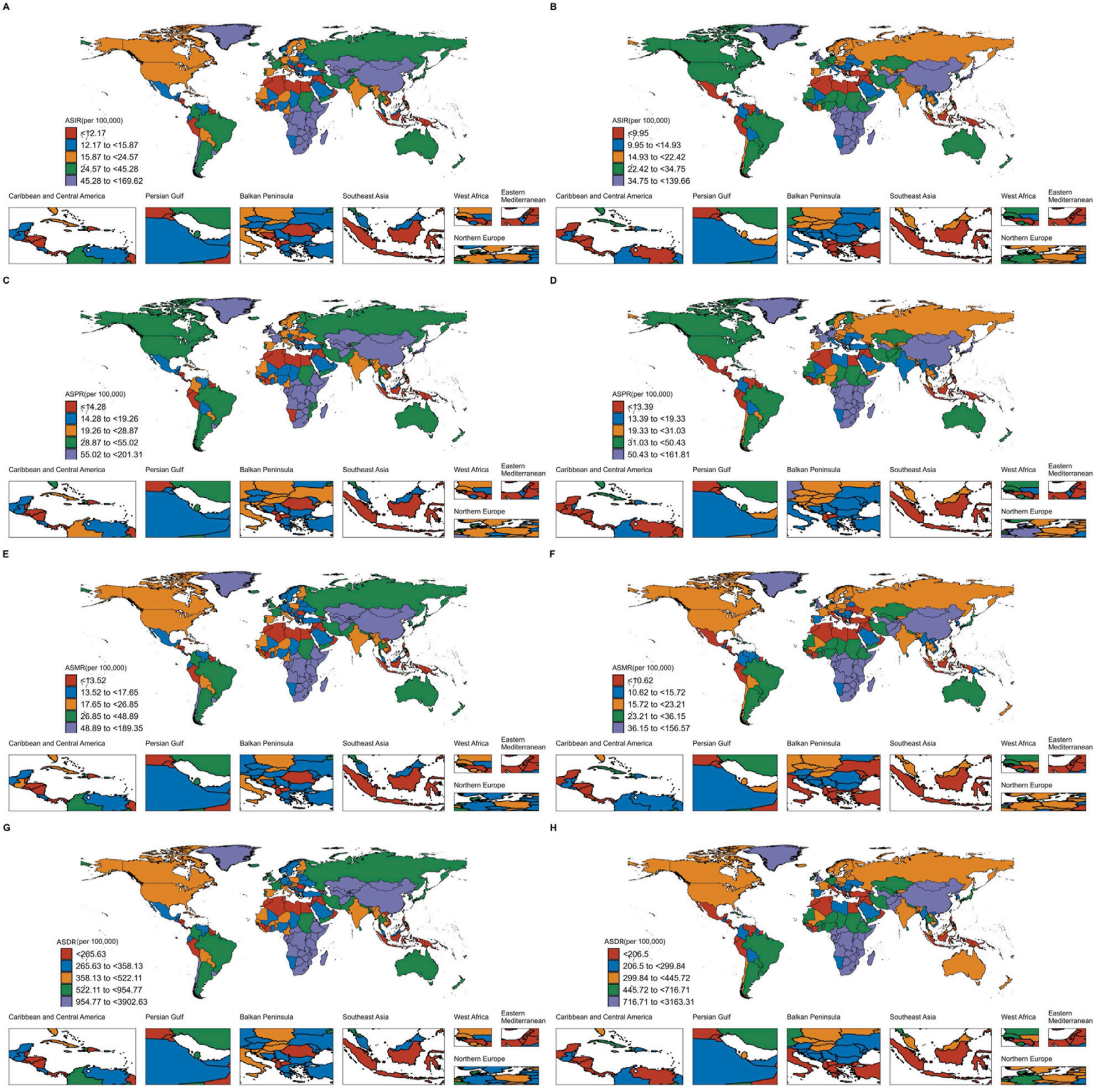
Age standardised in Incidence, Prevalence, Mortality and DALYs of EC in 204 countries and territories. **(A)** ASIR of EC in 204 countries and territories in 1990; **(B)** ASIR of EC in 204 countries and territories in 2021; **(C)** ASPR of EC in 204 countries and territories in 1990; **(D)** ASPR of EC in 204 countries and territories in 2021; **(E)** ASMR of EC in 204 countries and territories in 1990; **(F)** ASMR of EC in 204 countries and territories in 2021; **(G)** Age-standardized DALYs rate of EC in 204 countries and territories in 1990; **(H)** Age-standardized DALYs rate of EC in 204 countries and territories in 2021.

Geographically, from 1990 to 2021, the global age-standardized prevalence rate of esophageal cancer (EC) in individuals over 60 showed a general declining trend. Although the absolute number of cases remained at approximately 0.06 million, the average annual percentage change (AAPC) was recorded at −0.04 (95% UI: 0.17 to 0.09), indicating a slight overall decrease. Many regions experienced a reduction in the age-standardized prevalence rate, with East Asia being a significant contributor to the majority of EC cases in this age group. Despite this, East Asia witnessed a notable decline in its age-standardized prevalence rate from 0.16 million to 0.13 million, with an AAPC of −0.61 (95% UI: 0.79 to −0.42). Central Asia also experienced a pronounced decrease, with the rate plummeting from 0.09 million in 1990 to 0.03 million in 2021, marked by an AAPC of −3.31 (95% UI: 3.96 to −2.65). While the overall global trend shows a decrease in the age-standardized prevalence rate, certain regions such as Southern Sub-Saharan Africa, High-income Asia Pacific, Western Europe, Australasia, High-income North America, Western Sub-Saharan Africa, North Africa and the Middle East, and Central Europe also reported declining rates (Tables 3; Figure 2).

**TABLE 3:** Age standardised and AAPC in Incidence and Prevalence of EC in people aged ≥60 years at global and regional level, 1990-2021.

Location	Age-standardized incidence rate (per 100,000)			Age-standardized prevalence rate (per 100,000)		
	1990 (95% UI)	2021 (95% UI)	AAPC (95% UI)	1990 (95% UI)	2021 (95% UI)	AAPC (95% UI)
Global	47.25 (52.04–41.49)	39.06 (44.33–33.93)	−0.63 (-0.77 to −0.49)	60.93 (66.95–53.62)	60.21 (68.05–52.65)	−0.04 (-0.17 to 0.09)
High SDI	29.34 (30.54–27.62)	29.50 (31.22–26.88)	0.00 (-0.16–0.17)	47.00 (49.03–44.45)	61.55 (65.24–56.39)	0.84 (0.60–1.09)
High-middle SDI	58.02 (65.35–50.02)	51.30 (62.83–41.59)	−0.40 (-0.63 to −0.17)	71.03 (80.01–61.14)	77.54 (95.28–62.67)	0.29 (0.07–0.52)
Middle SDI	75.67 (87.65–61.89)	49.43 (59.59–40.71)	−1.38 (-1.53 to −1.22)	91.68 (106.53–74.21)	68.63 (83.14–56.17)	−0.91 (-1.05 to −0.78)
Low-middle SDI	21.48 (24.97–18.91)	19.08 (22.22–16.97)	−0.37 (-0.54 to −0.20)	24.80 (28.72–21.92)	22.70 (26.36–20.24)	−0.27 (-0.43 to −0.12)
Low SDI	35.92 (40.90–29.78)	30.06 (35.02–25.31)	−0.56 (-0.67 to −0.44)	40.91 (46.52–33.89)	34.92 (40.71–29.38)	−0.50 (-0.55 to −0.45)
East Asia	133.88 (155.16–108.45)	90.25 (110.36–72.10)	−1.28 (-1.48 to −1.08)	159.64 (185.74–128.40)	131.71 (161.63–105.02)	−0.61 (-0.79 to −0.42)
Eastern Sub-Saharan Africa	73.47 (85.85–59.89)	61.44 (74.50–50.56)	−0.58 (-0.66 to −0.50)	83.31 (97.22–67.88)	71.34 (86.79–58.72)	−0.51 (-0.55 to −0.46)
Central Asia	72.17 (77.43–66.74)	26.32 (29.52–23.34)	−3.33 (-4.11 to −2.54)	85.67 (91.71–79.64)	30.99 (34.72–27.52)	−3.31 (-3.96 to −2.65)
Central Sub-Saharan Africa	57.04 (75.03–41.48)	45.91 (62.35–32.57)	−0.71 (-0.83 to −0.60)	64.27 (84.67–46.49)	52.90 (71.39–37.62)	−0.64 (-0.75 to −0.52)
Southern Sub-Saharan Africa	53.40 (64.30–45.40)	57.42 (64.60–50.47)	0.22 (-0.10–0.53)	61.85 (74.60–52.59)	65.69 (73.80–58.02)	0.19 (-0.21–0.59)
Southern Latin America	42.76 (47.63–38.00)	24.36 (27.68–21.08)	−1.80 (-2.01 to −1.58)	49.27 (54.79–44.02)	30.25 (34.42–26.31)	−1.49 (-1.77 to −1.21)
High-income Asia Pacific	38.17 (40.55–35.33)	35.52 (38.43–31.65)	−0.28 (-0.49 to −0.06)	84.37 (91.74–76.84)	108.05 (117.14–96.57)	0.75 (0.54–0.96)
Tropical Latin America	34.41 (36.52–31.79)	25.36 (27.23–22.95)	−0.93 (-1.09 to −0.77)	40.33 (42.67–37.51)	32.01 (34.27–29.21)	−0.68 (-0.87 to −0.50)
Western Europe	25.96 (27.26–24.29)	25.00 (26.83–22.56)	−0.13 (-0.36 to 0.10)	35.90 (37.75–33.79)	47.67 (51.54–43.06)	0.92 (0.63–1.21)
Australasia	27.93 (31.34–24.56)	25.30 (29.39–21.16)	−0.37 (-0.84 to 0.10)	42.03 (47.49–36.91)	43.73 (51.22–36.71)	0.08 (-0.37–0.54)
Caribbean	22.38 (24.65–20.25)	18.97 (21.91–16.21)	−0.44 (-0.63 to −0.26)	26.39 (29.03–23.91)	24.51 (28.38–20.88)	−0.13 (-0.34 to 0.08)
High-income North America	22.69 (23.64–21.29)	24.61 (25.99–22.50)	0.20 (0.11–0.29)	34.62 (35.98–32.73)	41.22 (43.54–38.05)	0.53 (0.37–0.68)
Eastern Europe	21.38 (22.25–20.40)	15.20 (16.59–13.71)	−0.99 (-1.37 to −0.60)	26.81 (27.87–25.66)	21.44 (23.44–19.37)	−0.59 (-0.97 to −0.21)
South Asia	20.14 (24.54–17.20)	17.53 (21.23–15.26)	−0.40 (-0.67 to −0.12)	23.23 (28.18–19.98)	20.79 (25.11–18.16)	−0.35 (-0.60 to −0.10)
Central Latin America	15.70 (16.65–14.66)	9.21 (10.43–8.07)	−1.78 (-2.25 to −1.31)	17.60 (18.66–16.51)	10.99 (12.48–9.63)	−1.58 (-2.00 to −1.17)
Western Sub-Saharan Africa	15.11 (18.15–12.31)	24.82 (29.55–18.36)	1.62 (1.51–1.73)	17.28 (20.84–13.99)	28.49 (34.09–20.96)	1.63 (1.55–1.71)
North Africa and Middle East	14.57 (16.95–11.81)	11.93 (13.48–10.19)	−0.62 (-0.74 to −0.51)	17.61 (20.39–14.28)	17.82 (20.18–15.24)	0.04 (-0.07–0.15)
Southeast Asia	14.25 (17.05–11.74)	13.01 (15.30–10.94)	−0.32 (-0.40 to −0.24)	16.95 (20.31–13.95)	16.90 (19.96–14.14)	−0.02 (-0.10 to 0.06)
Central Europe	13.67 (14.41–12.87)	14.14 (15.36–12.81)	0.12 (-0.30–0.54)	16.26 (17.13–15.34)	19.19 (20.90–17.42)	0.57 (0.18–0.96)
Andean Latin America	12.18 (14.51–10.14)	8.83 (11.21–6.95)	−0.98 (-1.73 to −0.23)	13.64 (16.24–11.35)	10.21 (13.07–7.99)	−0.76 (-0.97 to −0.54)
Oceania	12.05 (16.37–8.92)	10.49 (13.94–8.02)	−0.43 (-0.48 to −0.38)	13.46 (18.34–9.91)	12.07 (16.03–9.23)	−0.37 (-0.41 to −0.32)
Male	70.06 (78.55–62.37)	62.61 (72.78–53.38)	−0.38 (-0.52 to −0.24)	88.99 (99.63–79.61)	93.34 (107.92–80.33)	0.14 (0.01–0.28)
Female	29.13 (33.93–20.51)	19.49 (23.22–14.18)	−1.28 (-1.36 to −1.21)	37.72 (43.86–26.46)	31.79 (38.21–22.76)	−0.52 (-0.63 to −0.41)
60–64 years	36.19 (40.20–32.01)	23.03 (26.25–20.39)	−1.43 (−1.63 to −1.24)	58.78 (65.06–52.16)	44.38 (50.35–39.44)	−0.93 (−1.14 to −0.72)
65–69 years	45.81 (60.72–39.70)	34.28 (39.00–29.78)	−0.94 (−1.10 to −0.78)	66.53 (73.47–57.68)	61.24 (69.49–53.49)	−0.28 (−0.49 to −0.08)
70–74 years	57.57 (63.64–50.48)	45.45 (61.67–39.69)	−0.73 (−0.84 to −0.63)	73.08 (80.36–64.31)	75.04 (84.46–65.63)	0.13 (−0.00–0.26)
75–79 years	55.74 (61.13–49.57)	52.61 (59.76–45.68)	−0.20 (−0.57 to 0.17)	60.05 (65.45–53.64)	72.87 (82.56–63.84)	0.58 (0.13–1.02)
80–84 years	51.67 (55.74–45.80)	55.72 (62.74–47.96)	0.25 (−0.17–0.67)	47.92 (51.38–42.59)	67.50 (75.70–58.55)	1.14 (0.86–1.42)
85–89 years	53.29 (57.48–46.33)	64.77 (72.99–54.90)	0.63 (0.43–0.82)	39.70 (42.71–34.26)	57.73 (64.82–48.58)	1.23 (1.01–1.45)
90–94 years	45.63 (49.50–28.66)	52.77 (69.44–43.28)	0.47 (0.26–0.68)	29.09 (31.61–24.53)	39.21 (44.30–31.55)	0.99 (0.80–1.18)
95+ years	32.61 (36.68–25.62)	41.73 (48.08–31.06)	0.81 (0.63–0.99)	16.71 (18.80–13.11)	22.63 (26.12–16.78)	1.00 (0.82–1.18)

In terms of DALYs, the changes were less pronounced compared to those seen in incidence and mortality rates. The age-standardized DALYs associated with EC over 60 decreased by 28.85%, from 1,040 per 100,000 in 1990 to 740 per 100,000 in 2021, with an average annual trend of −0.99% (Tables 3, 4).

**TABLE 4:** Age standardised and AAPC in Mortality and DALYs of EC in people aged ≥60 years at global and regional level, 1990-2021.

Location	Age-standardized mortality rate (per 100,000)			Age-standardized DALYs rate (per 100,000)		
	1990 (95% UI)	2021 (95% UI)	AAPC (95% UI)	1990 (95% UI)	2021 (95% UI)	AAPC (95% UI)
Global	50.44 (55.53–44.24)	38.25 (43.41–33.22)	−0.91 (-1.07 to −0.76)	1,040.22 (1,149.30–913.01)	739.86 (841.06–645.41)	−1.12 (-1.26 to −0.97)
High SDI	28.34 (29.54–26.68)	24.98 (26.49–22.72)	−0.42 (-0.60 to −0.24)	570.41 (593.05–542.94)	483.60 (510.91–447.41)	−0.54 (-0.72 to −0.36)
High-middle SDI	63.13 (71.01–54.38)	49.80 (60.56–40.59)	−0.78 (-1.02 to −0.55)	1,294.78 (1,459.15–1,116.70)	962.32 (1,177.80–784.67)	−0.97 (-1.23 to −0.71)
Middle SDI	82.97 (95.86–68.10)	50.40 (60.37–41.55)	−1.61 (-1.78 to −1.44)	1702.87 (1977.98–1,392.24)	955.41 (1,147.18–788.64)	−1.88 (-2.04 to −1.72)
Low-middle SDI	24.06 (28.06–21.18)	21.22 (24.81–18.87)	−0.39 (-0.59 to −0.19)	488.22 (566.94–431.88)	422.18 (491.35–376.81)	−0.46 (-0.62 to −0.30)
Low SDI	40.21 (45.80–33.34)	33.77 (39.42–28.44)	−0.54 (-0.66 to −0.43)	827.17 (940.31–686.05)	675.97 (788.11–569.31)	−0.63 (-0.69 to −0.58)
East Asia	147.59 (170.72–119.80)	89.05 (108.62–71.05)	−1.63 (-1.85 to −1.42)	2,955.52 (3,439.17–2,397.19)	1,671.14 (2052.44–1,329.33)	−1.85 (-2.05 to −1.64)
Eastern Sub-Saharan Africa	82.37 (96.41–67.19)	69.00 (83.61–56.86)	−0.57 (-0.65 to −0.49)	1,688.34 (1971.98–1,378.62)	1,381.79 (1,674.55–1,141.36)	−0.64 (-0.72 to −0.56)
Central Asia	80.55 (86.48–74.36)	29.34 (32.89–26.01)	−3.33 (-4.13 to −2.53)	1,647.99 (1764.58–1,529.79)	587.62 (659.17–521.16)	−3.38 (-4.04 to −2.71)
Central Sub-Saharan Africa	64.07 (84.52–46.62)	51.62 (70.60–36.54)	−0.71 (-0.82 to −0.60)	1,311.23 (1726.06–952.94)	1,035.06 (1,397.53–734.72)	−0.78 (-0.90 to −0.66)
Southern Sub-Saharan Africa	60.38 (72.86–51.32)	64.37 (72.43–56.54)	0.19 (-0.11–0.50)	1,178.01 (1,416.05–1,003.24)	1,264.59 (1,420.84–1,116.27)	0.22 (-0.20–0.64)
Southern Latin America	48.08 (53.59–42.68)	26.45 (30.06–22.82)	−1.91 (-2.13 to −1.69)	901.57 (1,005.01–804.97)	487.80 (554.72–423.85)	−1.90 (-2.19 to −1.61)
High-income Asia Pacific	31.03 (32.95–28.69)	23.07 (24.89–20.56)	−1.02 (-1.10 to −0.94)	614.97 (652.46–574.10)	439.42 (473.58–398.20)	−1.17 (-1.26 to −1.09)
Tropical Latin America	38.55 (40.96–35.58)	27.66 (29.72–24.95)	−1.01 (-1.17 to −0.85)	766.04 (811.51–713.67)	561.10 (600.32–511.97)	−0.95 (-1.13 to −0.76)
Western Europe	26.82 (28.17–25.04)	22.56 (24.14–20.37)	−0.57 (-0.87 to −0.26)	529.84 (555.37–499.70)	439.37 (468.18–403.00)	−0.62 (-0.88 to −0.36)
Australasia	27.90 (31.33–24.55)	23.76 (27.65–19.86)	−0.54 (-0.72 to −0.37)	539.75 (604.66–477.31)	434.57 (504.70–367.76)	−0.73 (-0.89 to −0.58)
Caribbean	25.03 (27.60–22.65)	20.40 (23.58–17.44)	−0.57 (-0.76 to −0.38)	478.26 (528.33–432.85)	410.00 (474.61–349.56)	−0.42 (-0.61 to −0.22)
High-income North America	21.56 (22.48–20.20)	22.20 (23.47–20.29)	0.06 (-0.10–0.22)	443.82 (460.62–421.09)	435.54 (457.76–405.09)	−0.09 (-0.24 to 0.06)
Eastern Europe	22.86 (23.76–21.82)	15.31 (16.69–13.85)	−1.19 (-1.69 to −0.70)	481.15 (499.91–461.53)	333.60 (364.88–301.58)	−1.07 (-1.55 to −0.58)
South Asia	22.54 (27.60–19.19)	19.51 (23.69–17.00)	−0.39 (-0.63 to −0.15)	460.54 (561.12–394.98)	386.74 (467.18–338.10)	−0.56 (-0.81 to −0.31)
Central Latin America	18.03 (19.12–16.81)	10.28 (11.61–9.00)	−1.86 (-2.33 to −1.40)	328.97 (348.48–308.85)	189.84 (215.52–166.50)	−1.84 (-2.27 to −1.41)
Western Sub-Saharan Africa	16.99 (20.39–13.89)	27.99 (33.29–20.79)	1.63 (1.51–1.74)	343.42 (414.89–279.08)	550.02 (657.63–407.99)	1.54 (1.45–1.62)
North Africa and Middle East	16.39 (19.09–13.29)	13.18 (14.90–11.23)	−0.67 (-0.79 to −0.56)	322.34 (374.60–260.84)	242.73 (274.94–207.56)	−0.90 (-1.00 to −0.80)
Southeast Asia	15.80 (18.84–13.01)	13.84 (16.27–11.67)	−0.46 (-0.54 to −0.38)	321.73 (384.08–264.54)	275.35 (324.50–232.57)	−0.51 (-0.62 to −0.40)
Central Europe	15.19 (16.01–14.30)	14.99 (16.28–13.59)	0.01 (-0.31–0.34)	307.11 (323.55–290.00)	317.25 (344.61–288.47)	0.12 (-0.29–0.54)
Andean Latin America	13.98 (16.70–11.65)	9.97 (12.64–7.87)	−1.04 (-1.78 to −0.30)	259.57 (310.85–216.06)	178.20 (226.83–140.02)	−1.06 (-1.27 to −0.85)
Oceania	13.66 (18.56–10.21)	11.80 (15.75–9.04)	−0.48 (-0.54 to −0.41)	267.00 (363.94–197.76)	229.90 (307.58–176.34)	−0.50 (-0.55 to −0.46)
Male	75.08 (83.99–66.76)	61.88 (71.83–52.83)	−0.64 (-0.80 to −0.49)	1,551.18 (1746.87–1,375.35)	1,191.5 (1,387.99–1,015.32)	−0.87 (-1.02 to −0.73)
Female	31.2 (36.29–21.95)	18.92 (22.50–13.97)	−1.59 (-1.78 to −1.40)	619.00 (725.28–424.56)	352.09 (416.73–263.18)	−1.79 (-1.96 to −1.62)
60–64 years	35.20 (39.13–31.10)	20.37 (23.21–18.06)	−1.76 (−1.99 to −1.54)	1,026.16 (1,140.95–906.94)	594.12 (677.77–527.23)	−1.72 (−1.96 to −1.47)
65–69 years	46.20 (51.17–40.01)	30.97 (35.33–26.90)	−1.31 (−1.50 to −1.13)	1,134.65 (1,257.55–984.77)	761.44 (867.27–660.90)	−1.29 (−1.46 to −1.12)
70–74 years	60.84 (67.32–52.98)	42.48 (48.24–37.08)	−1.13 (−1.23 to −1.03)	1,229.84 (1,363.05–1,072.34)	860.29 (979.26–752.09)	−1.12 (−1.24 to −1.01)
75–79 years	62.46 (68.42–55.60)	53.20 (60.47–46.28)	−0.53 (−0.92 to −0.14)	1,012.82 (1,109.06–900.44)	862.33 (979.69–751.32)	−0.54 (−0.92 to −0.15)
80–84 years	61.67 (66.63–55.06)	60.16 (67.87–51.81)	−0.08 (−0.49 to 0.33)	783.57 (847.84–700.68)	762.55 (860.31–657.14)	0.09 (−0.47 to 0.30)
85–89 years	67.27 (72.69–68.51)	74.51 (83.57–63.50)	0.31 (0.10–0.53)	680.62 (735.78–592.68)	751.01 (842.61–640.57)	0.30 (0.09–0.51)
90–94 years	64.82 (70.17–54.93)	71.45 (80.35–58.95)	0.31 (0.09–0.52)	568.59 (615.89–482.92)	626.91 (705.01–516.10)	0.21 (0.09–0.52)
95+ years	49.07 (55.10–38.63)	57.70 (66.19–43.00)	0.54 (0.35–0.73)	406.50 (455.92–320.69)	473.96 (544.82–353.48)	0.51 (0.32–0.71)

### The Social Demographic Index (SDI) analysis

**FIGURE 3:**
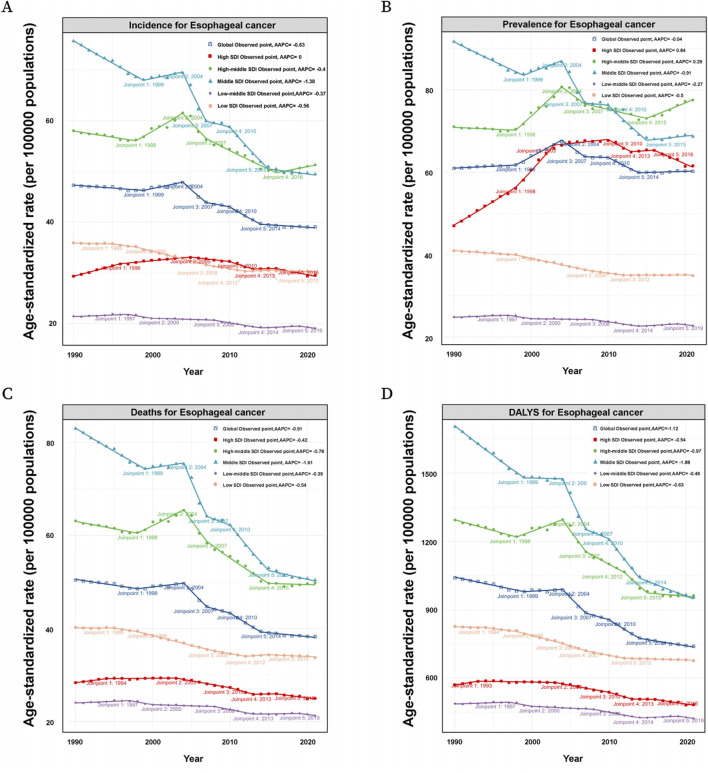
**(A)** Incidence of patients by SDI (Global, High SDI, High-middle SDI, Middle SDI, Low-middle SDI, Low SDI) in EC at the global level based on the joinpoint regression analysis model. **(B)** Prevalence of patients by SDI (Global, High SDI, High-middle SDI, Middle SDI, Low-middle SDI, Low SDI) in EC at the global level based on the joinpoint regression analysis model. **(C)** Mortality of patients by SDI (Global, High SDI, High-middle SDI, Middle SDI, Low-middle SDI, Low SDI) in EC at the global level based on the joinpoint regression analysis model. **(D)** DALYs of patients by SDI (Global, High SDI, High-middle SDI, Middle SDI, Low-middle SDI, Low SDI) in EC at the global level based on the joinpoint regression analysis model.

**FIGURE 4:**
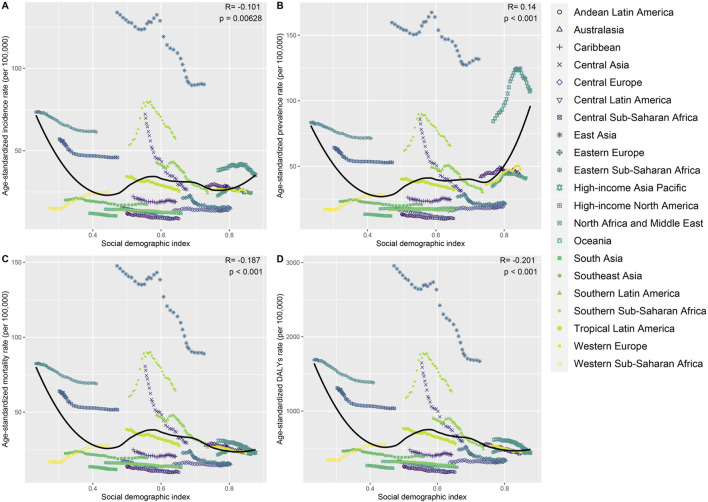
The ASIR, ASPR, ASMR and ASDR in patients of different regions. **(A)** The ASIR of patients by SDI (Global, High SDI, High-middle SDI, Middle SDI, Low-middle SDI, Low SDI) in EC at the global level based on the joinpoint regression analysis model. **(B)** The ASPR of patients by SDI (Global, High SDI, High-middle SDI, Middle SDI, Low-middle SDI, Low SDI) in EC at the global level based on the joinpoint regression analysis model. **(C)** The ASMR of patients by SDI (Global, High SDI, High-middle SDI, Middle SDI, Low-middle SDI, Low SDI) in EC at the global level based on the joinpoint regression analysis model. **(D)** The ASDR of patients by SDI (Global, High SDI, High-middle SDI, Middle SDI, Low-middle SDI, Low SDI) in EC at the global level based on the joinpoint regression analysis model.

Based on the Social Demographic Index (SDI) analysis, significant variations in Age-standardized Incidence Rate (ASIR), Age-standardized Prevalence Rate (ASPR), DALYs Rate, and Age-standardized Deaths Rate for EC among patients aged 60 and over have been observed globally from 1990 to 2021. These variations reflect differing trends across various SDI categories (Figures 2–4).

Globally, the ASIR of EC for those over 60 years showed fluctuating trends between 1990 and 2021. Specifically, from 1990 to 1999, the APC was −0.25 (95% UI: 0.34 to −0.16), followed by a brief increase from 1999 to 2004 with an APC of 0.70 (95% UI: 0.39–1.01). The ASIR then declined again from 2004 to 2007 with an APC of −2.89 (95% UI: 3.78 to −1.98). Subsequent years showed minor variations with an overall decreasing trend, culminating in a slight decline from 2014 to 2021, with an APC of −0.25 (95% UI: 0.41 to −0.10). In high SDI regions, a more pronounced increase in ASIR was observed during the early years (1990-1999), with an APC of 1.38 (95% UI: 1.22–1.54). However, this was followed by a mixed trend, with a notable decline between 2007 and 2010 (APC: 1.58, 95% UI: 2.68 to −0.46) and a further decrease in the following years. In high-middle SDI regions, the trends were more varied, with an initial decrease from 1990 to 1999, followed by a notable increase between 1999 and 2004 (APC: 1.55, 95% UI: 1.11–1.99). However, the subsequent period saw declines, particularly between 2004 and 2007 (APC: 2.75, 95% UI: 4.63 to −0.84). In middle SDI regions, a significant initial decrease was observed from 1990 to 1999, with an APC of −1.20 (95% UI: 1.31 to −1.09). Although there was a brief increase between 1999 and 2004 (APC: 0.47, 95% UI: 0.10–0.84), the trend again turned negative, especially between 2004 and 2007 (APC: 4.87, 95% UI: 5.91 to −3.83). Low-middle and low SDI regions generally showed declining ASIRs over the years, with occasional slight increases, such as in the low-middle SDI group between 1990 and 1997 (APC: 0.30, 95% UI: 0.12–0.47), but with an overall downward trend by 2021 (Figure 3A).

The global ASPR for EC in patients over 60 also displayed fluctuations (Figure 3B). Between 1990 and 1998, the ASPR increased slightly with an APC of 0.15 (95% UI: 0.05–0.26), followed by a more substantial rise between 1998 and 2004 (APC: 1.57, 95% UI: 1.37–1.78). However, this was followed by a period of decline, particularly from 2004 to 2007 (APC: 2.04, 95% UI: 2.89 to −1.19), with more modest changes thereafter. In high SDI regions, the ASPR rose significantly in the early years, particularly between 1990 and 1998 (APC: 2.25, 95% UI: 2.09–2.41), and between 1998 and 2003 (APC: 3.38, 95% UI: 2.89–3.87). However, a sharp decline followed from 2010 to 2013 (APC: 1.50, 95% UI: 3.14 to 0.17), with a continuation of this trend in subsequent years. For high-middle SDI regions, the trends varied, with initial stability followed by significant increases, particularly between 1998 and 2004 (APC: 2.36, 95% UI: 1.92–2.80). This was followed by fluctuations, with a slight increase from 2015 to 2021 (APC: 1.00, 95% UI: 0.53–1.48). Middle, low-middle, and low SDI regions generally showed declining ASPRs, with occasional slight increases. For example, in low SDI regions, the ASPR decreased steadily from 1998 to 2021, with a brief period of stabilization from 2012 to 2021 (APC: 0.00, 95% UI: 0.06–0.07).

Globally, the age-standardized death rate for EC among patients over 60 has demonstrated a declining trend, particularly from 1990 to 1998 (APC: 0.51, 95% UI: 0.63 to −0.38) and again from 2004 to 2007 (APC: 3.56, 95% UI: 4.56 to −2.54). High SDI regions initially experienced an increase followed by a decline, while high-middle SDI regions exhibited a more complex pattern, marked by significant declines, particularly between 2004 and 2007 (APC: 3.56, 95% UI: 4.56 to −2.54) (Figure 3C). Additionally, the global age-standardized DALYs rate for EC showed a general decline, especially from 1990 to 1999 (APC: 0.70, 95% UI: 0.80 to −0.60), and again more sharply between 2004 and 2007 (APC: 3.63, 95% UI: 4.55 to −2.70). This trend continued with minor variations, indicating an overall reduction in the disease burden over time. High SDI regions experienced a fluctuating DALYs rate, with an initial slight increase followed by a decline, particularly between 2003 and 2010 (APC: 1.09, 95% UI: 1.28 to −0.89). High-middle SDI regions displayed a similar pattern, with a significant decline between 2004 and 2007 (APC: 3.77, 95% UI: 5.39 to −2.13). Middle, low-middle, and low SDI regions generally followed a declining trend in DALYs, with middle SDI regions showing the most pronounced decrease from 2004 to 2007 (APC: 5.28, 95% UI: 6.33 to −4.22) (Figure 3D).

### Global trends by age subgroup

**FIGURE 5:**
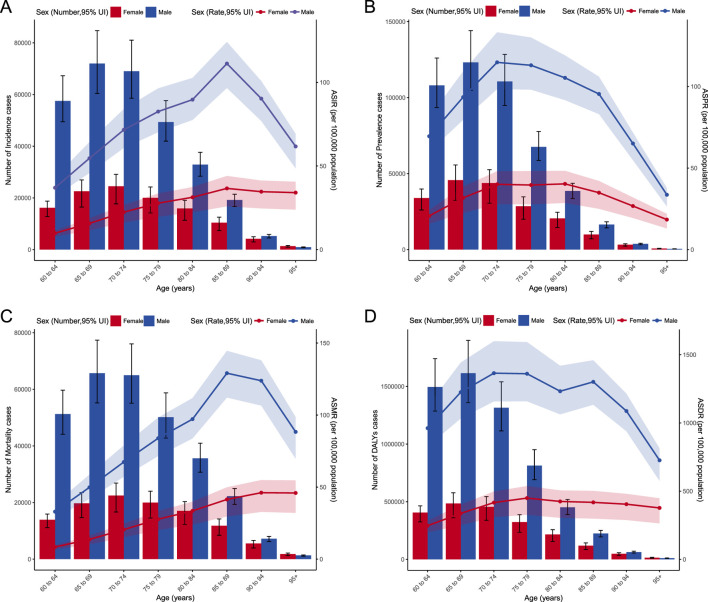
**(A)** Incidence and ASIR of EC by age and sex with analyses; **(B)** Prevalence and ASPR of EC by age and sex with analyses; **(C)** Mortality and ASMR of EC by age and sex with analyses; **(D)** DALYs and ASDR of EC by age and sex with analyses.

In age subgroup analyses from 1990, the highest incidence of EC patients over 60 was in the 60-64 age group with approximately 58.1 thousand cases, followed by the 65-69 age group with about 56.6 thousand cases. The 70–74 group had 48.7 thousand patients, 75–79 had 34.3 thousand, 80–84 had 18.2 thousand, 85–89 had 8.0 thousand, 90–94 had 2.0 thousand, and those 95 and above had 0.3 thousand. In 2021, there was a notable shift in the distribution of EC patients aged 60 and over. The 65-69 age group became the largest with approximately 94.6 thousand cases, followed closely by the 70–74 group with 93.5 thousand cases. The 60–64 group had 73.7 thousand patients, 75–79 had 69.3 thousand, 80–84 had 48.8 thousand, 85–89 had 29.6 thousand, 90–94 had 9.4 thousand, and those 95 and above had 2.2 thousand (Figure 5).

**FIGURE 6:**
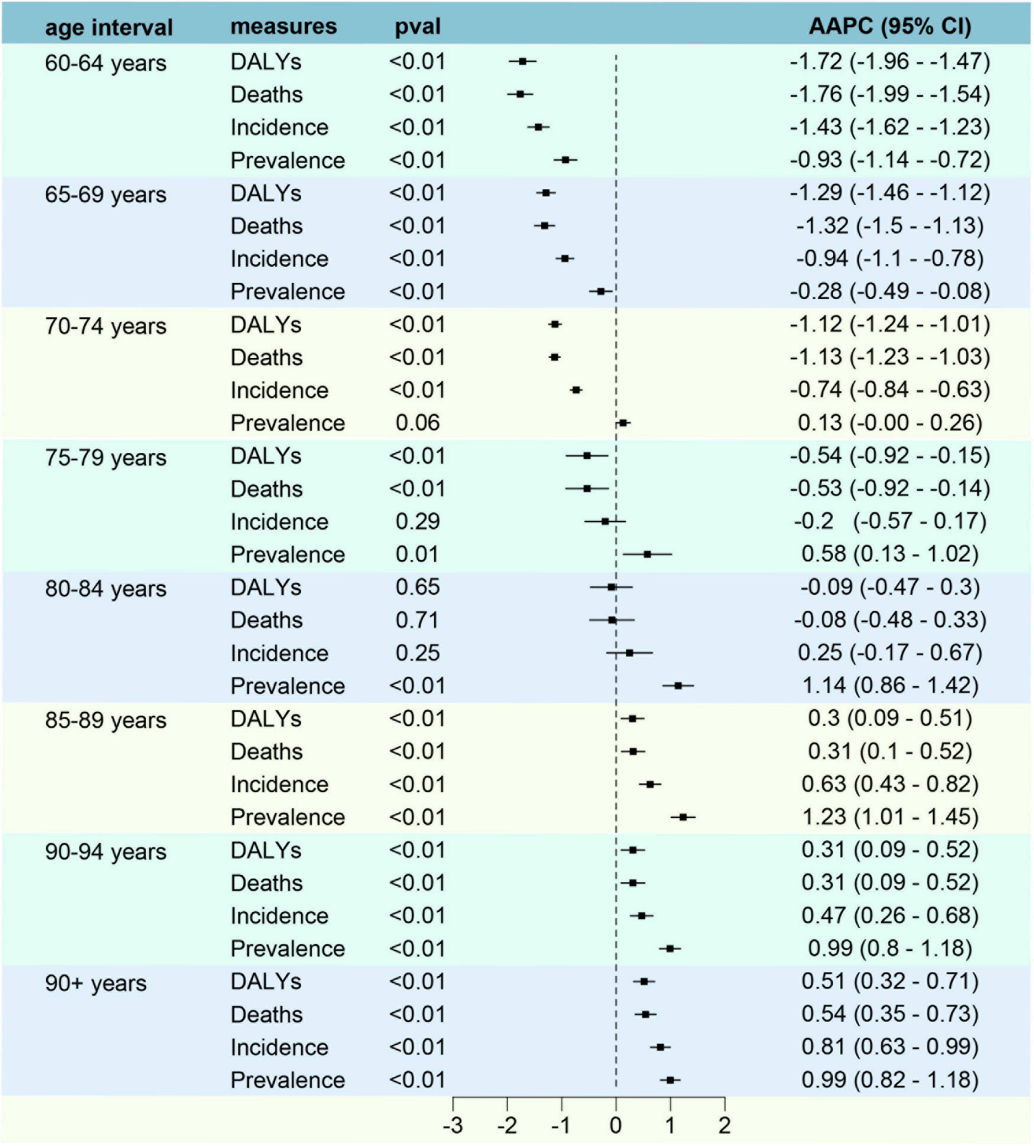
AAPC in Incidence, Prevalence, Mortality and DALYs of EC by age and sex with analyses.

The analysis of ASIR from 1990 to 2021 reveals notable trends across different age groups. In 1990, the highest incidence rate was observed in the 70-74 age group, with an ASIR of 57.57 per 100,000. Over the years, this age group continued to show significant incidence rates, maintaining the highest ASIR of 45.45 per 100,000 in 2021. A closer look at the AAPC indicates varying trends across age groups. Younger age groups (60-64 and 65-69) experienced a decline in ASIR, with AAPCs of −1.43 and −0.94, respectively. Conversely, older age groups, particularly those aged 80-84 and above, showed increasing AAPCs, with the highest increase observed in individuals aged 95 and above (AAPC of 0.81) (Figures 5, 6). Despite this, East Asia witnessed a notable decline in its age-standardized prevalence rate from 159.64 per 100,000 in 1990 to 134.21 per 100,000 in 2021, while the absolute number of prevalent cases decreased from approximately 0.16 million to 0.13 million, with an AAPC of −0.61 (95% CI: 0.79 to −0.42).

### Risk factors and death analyses

**FIGURE 7:**
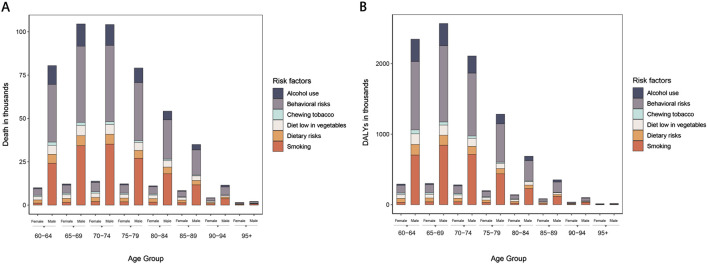
Risk factors of EC by age and sex with analyses.

Behavioral risks, including smoking and alcohol use, are prominent factors contributing to the burden of EC among patients over the age of 60. The data highlights that behavioral risks were responsible for 0.23 million deaths, leading to 4.49 million DALYs in this population. Smoking alone accounted for 0.17 million deaths and 3.27 million DALYs, while alcohol use contributed to 0.06 million deaths and 1.19 million DALYs. These figures underscore the significant impact of lifestyle-related risk factors on the health outcomes of older adults with EC (Figure 7).

When examining the gender-specific data, the disparities become more pronounced. Among male patients over 60, behavioral risks were responsible for 0.20 million deaths, resulting in 3.97 million DALYs. Smoking, a predominant risk factor among men, led to 0.15 million deaths and 3.09 million DALYs, while alcohol use contributed to 0.05 million deaths and 1.12 million DALYs. This data indicates that men are disproportionately affected by these risk factors, reflecting the higher prevalence of smoking and alcohol consumption among older males. In contrast, female EC patients over 60 experienced lower but still significant impacts from these risks. Behavioral risks were linked to 0.05 million deaths and 0.52 million DALYs, with smoking responsible for 0.01 million deaths and 0.19 million DALYs (Figure 7).

### Trends of esophageal cancer from 1990 to 2050

**FIGURE 8:**
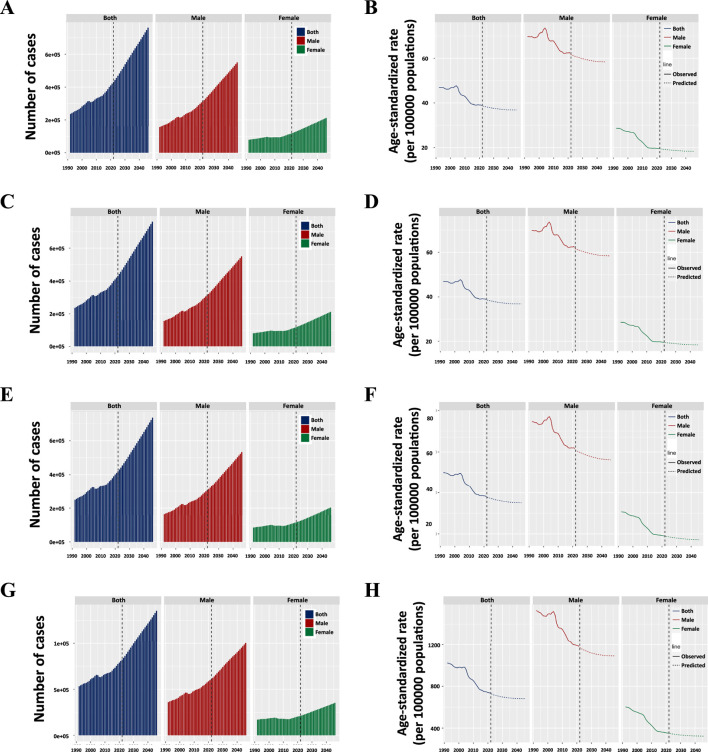
**(A)** Projected numbers and age-standardized rates (ASR) of incidence; **(B)** Projected numbers and age-standardized rates (ASR) of ASIR; **(C)** Projected numbers and age-standardized rates (ASR) of prevalence; **(D)** Projected numbers and age-standardized rates (ASR) of ASPR; (E) Projected numbers and age-standardized rates (ASR) of mortality; **(E)** Projected numbers and age-standardized rates (ASR) of ASMR; **(F)** Projected numbers and age-standardized rates (ASR) of ASPR; **(E)** Projected numbers and age-standardized rates (ASR) of DALYs; **(G)** Projected numbers and age-standardized rates (ASR) of ASDR.

This projected data underscores the ongoing global challenge posed by esophageal cancer, particularly among the aging population. Despite the declining ASR for incidence, mortality, and DALYs, the absolute numbers are expected to rise steadily due to the growing and aging population. By 2040, the incidence of esophageal cancer is anticipated to exceed 0.70 million, with prevalence surpassing 1.00 million by 2037. Similarly, mortality is expected to climb, with more than 0.70 million deaths projected by 2044, and DALYs are forecasted to reach over 13.00 million by the same year (Figure 8). Men are projected to remain the most affected group, with their incidence surpassing 0.40 million by 2031 and 0.50 million by 2042. Similarly, the prevalence in men is expected to exceed 0.70 million by 2036 and 0.80 million by 2043, while mortality is forecasted to exceed 0.40 million by 2033 and 0.50 million by 2043. The DALYs for men are expected to surpass 9.00 million by 2040 and 10.00 million by 2046. In contrast, women, though less affected, will also see significant increases, with incidence surpassing 0.20 million by 2044, prevalence exceeding 0.30 million by 2037, and mortality surpassing 0.20 million by 2046. The DALYs for women are projected to exceed 3.00 million by 2038 (Figure 8).

## Discussion

The data reveal a substantial increase in the incidence of EC among individuals over 60, which, although contrasted by a decrease in age-standardized rates, underscores a pressing burden on healthcare systems. This rise in absolute numbers can be attributed to the aging global population, a phenomenon that necessitates enhanced screening and prevention programs specifically tailored to this age group. The apparent disparity in incidence between genders, with males more significantly affected than females, suggests the need for targeted interventions considering behavioral and biological differences that may influence risk factors. The predominance of EC cases in East Asia and the significant reduction in the age-standardized prevalence rate within this region, despite an increase in total cases, could reflect improvements in healthcare access and early detection methods. The varying trends across different SDI categories highlight the influence of socioeconomic factors on EC incidence and outcomes. High SDI regions showed initial increases in incidence followed by declines, potentially reflecting better health literacy and access to medical care leading to early detection and effective treatment. Conversely, the fluctuations in lower SDI regions emphasize the need for global health initiatives to address inequalities in healthcare access and delivery.

The decline in DALYs and age-standardized mortality rates globally indicates improvements in treatment efficacy and possibly lifestyle changes that reduce the severity of EC. However, this positive trend is contrasted by the alarming increase in absolute numbers of cases and deaths attributed to EC, emphasizing the persistent challenge of managing chronic diseases in aging populations. The data on DALYs further illustrate the substantial burden of disease, which continues to require significant healthcare resources and patient care strategies. Projections of rising incidence and mortality rates up to 2050 present a critical opportunity for intervention. Additionally, the notable influence of behavioral risk factors, such as smoking and alcohol consumption, on EC mortality and DALYs underscores the urgent need for comprehensive public health campaigns and policies focused on reducing these behaviors. Research has demonstrated that patients who smoke and consume alcohol experience poorer survival outcomes following surgical treatment. These changes are especially relevant for males, who historically have higher rates of these behaviors, contributing to their increased incidence of EC (20,21).

Recent years have indeed seen a general decline in the incidence of EC, likely due to enhanced cancer prevention and control measures. However, in high SDI regions, while there has been an observed increase in ASIR, this trend may be attributed to the aging population rather than other behavioral factors. Higher average life expectancy and a more pronounced aging demographic in these regions could lead to an increase in cancer incidence, as older age is a known risk factor for many cancers. On the other hand, the slight decrease in ASIR in middle-high SDI regions could be reflective of effective healthcare interventions, early detection, and possibly a younger population structure compared to high SDI regions. Further studies focusing on behavioral factors could provide additional insights into these trends. These findings suggest a shift in disease burden towards the older populations over the past decades. The observed decline in ASIR among younger age groups could be attributed to advancements in early detection and preventative measures, while the increase in older age groups may reflect an aging population with longer life expectancies and improved healthcare access.

Current treatment modalities for EC primarily revolve around surgery, often combined with radiotherapy, chemotherapy, and increasingly, immunotherapy (22–27). Esophagectomy typically involve two-field or three-field lymphadenectomy (28–31). The advancements in minimally invasive surgical techniques and endoscopic technologies, along with precision radiotherapy, have significantly improved patient outcomes (32–35). However, despite these advancements, the prognosis for EC remains poor. A major factor contributing to this is the advanced stage at which most patients are diagnosed, often after proximal or distal lymph node metastasis has occurred, particularly in mediastinal, abdominal, or cervical regions (36). The prognosis is further compromised by the high prevalence of the disease in older populations, who are more susceptible to treatment-related complications (37–39). This advanced-stage diagnosis underscores the need for enhanced early detection and diagnostic strategies. Poor tolerability of elder patients for radical treatments is another reason. Thus, novel treatment modalities with fewer toxicities should be developed.

There are several limitations that should be acknowledged. Firstly, its retrospective nature means the data may be subject to biases in collection and reporting, potentially affecting the reliability of trend analyses. The global data used are derived from various sources with differing levels of accuracy and completeness, and discrepancies in diagnostic criteria, reporting practices, and healthcare infrastructure across regions may have influenced the observed trends. Additionally, while the study highlights the role of behavioral risk factors like smoking and alcohol use, it may not fully account for other potential confounders such as dietary habits, genetic predispositions, and environmental factors. The broad categorization of regions could oversimplify the complexity of regional differences in EC epidemiology, masking important local variations in disease patterns and healthcare practices. Projections of future trends are inherently uncertain and could be influenced by unforeseen changes in population demographics, healthcare advancements, or public health interventions. The study also does not differentiate between subtypes of EC, such as squamous cell carcinoma and adenocarcinoma, which may have distinct risk factors, epidemiological patterns, and treatment responses. Lastly, the use of aggregate data limits the ability to perform in-depth analyses of individual-level factors, such as patient comorbidities, treatment responses, and survival outcomes. These limitations suggest that while the findings of this study are valuable for understanding global trends in EC, caution should be exercised in interpreting the results, and further research is needed to address these gaps.

## Conclusion

The data highlight the evolving epidemiological characteristics of EC, particularly in individuals over 60, driven by demographic changes, socioeconomic factors, and lifestyle influences. Furthermore, international collaboration in research and data sharing is essential for a comprehensive understanding and effective management of the global burden of EC.

## Data Availability

Publicly available datasets were analyzed in this study. This data can be found here: https://vizhub.healthdata.org/gbd-results/result/c121d96d2fd53001376a8f1154f33b87.

## Abbreviations

EC, esophageal cancer; SCC, squamous cell carcinoma; ESD, endoscopic submucosal dissection; GBD, Global Burden of Disease; DALYs, disability‐adjusted life years; ASIR, Age-standardized incidence rates; AAPC, annual average percentage change; ASPR, Age‐standardized Prevalence Rate; UI, confidence interval; UI, uncertainty interval; ASRs, age-standardized rates; APCs, annual percentage changes; BAPC, Bayesian Age-Period-Cohort; ASMR, Age-standardized Mortality Rate; ASDR, Age-standardized DALYs rate.
